# Supplementary material for: Deep Hybrid Learning Prediction of Patient-Specific Quality Assurance in Radiotherapy: Implementation in Clinical Routine
Source: Diagnostics (Basel). 2023 Mar 2;13(5):943. doi: 10.3390/diagnostics13050943 (PMC10001389; doi:10.3390/diagnostics13050943)
Supplement: Supplementary file 1 [file diagnostics-13-00943-s001.zip › diagnostics-2202896-supplementary.pdf]

Supp Table 1:

Tumor location	Mean Gamma Threshold
Pelvis	0.37 ± 0.044
Breast	0.44 ± 0.055
H&N	0.43 ± 0.053
Brain	0.42 ± 0.061
Thorax	0.41 ± 0.051
Digestive	0.36 ± 0.040
Rachis	0.38 ± 0.043
Other	0.41 ± 0.061
Member	0.43 ± 0.052
Skin	0.46 ± 0.065
All	0.42 ± 0.056

Gamma mean threshold for each tumor location

Supp Table 2:

Machine learning models	Training score	Validation score
LinearDiscriminant	0.73	0.59
Ridge	0.73	0.59
Kneighbors	0.94	0.82
GaussianNB	0.68	0.64
DecisionTree	0.83	0.76
SVC	0.87	0.75
SGD	0.73	0.60
LinearRegression	0.24	0
RandomForestClassifier	1	0.90

ML classifier models training and validation score (for “all tumor location”)

Supp FIGURE 1:

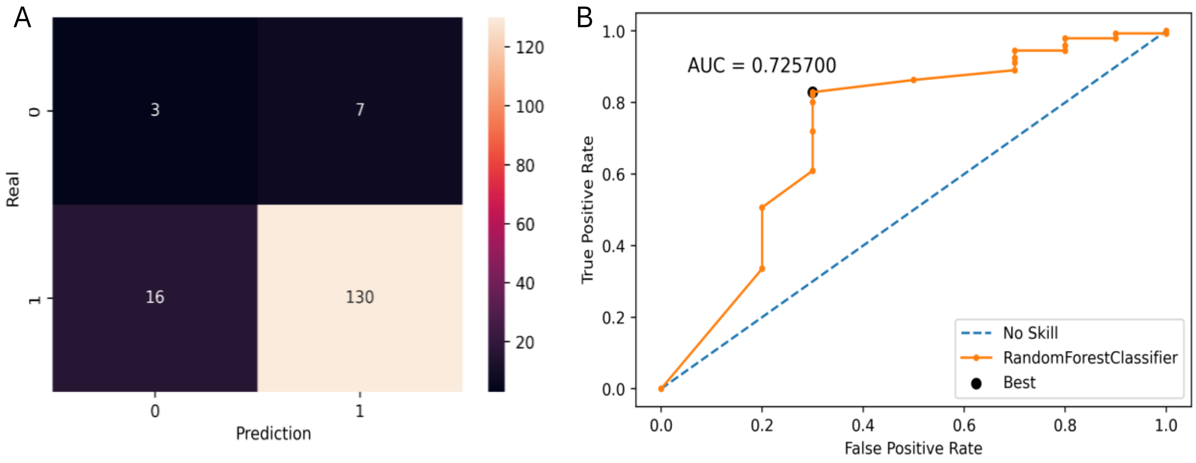

Confusion matrix (A) and ROC curve (B) for the global ML model applied to the Brain tumor RT plans

Supp Table 3 :

TP	TN	FP	FN	Sensitivity	Specificity
83.33 %	1.92 %	4.49 %	10.26 %	89.04 %	30 %

Performance for the global ML model applied to the Brain tumor RT plans

Supp FIGURE 2:

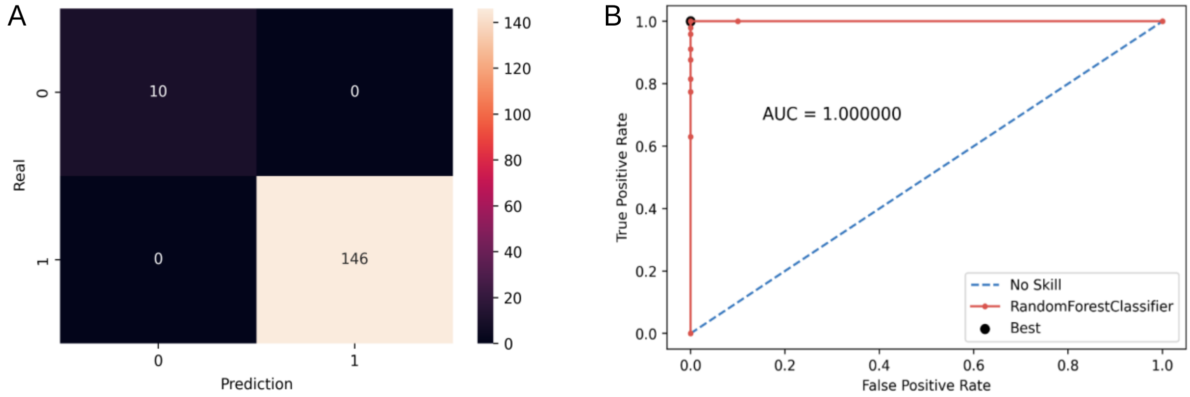

Confusion matrix (A) and ROC curve (B) the brain specific ML model

Supp Table 4 :

TP	TN	FP	FN	Sensitivity	Specificity
93.59 %	6.41 %	0 %	0 %	100 %	100 %

Performance of the brain specific ML model

Supp FIGURE 3:

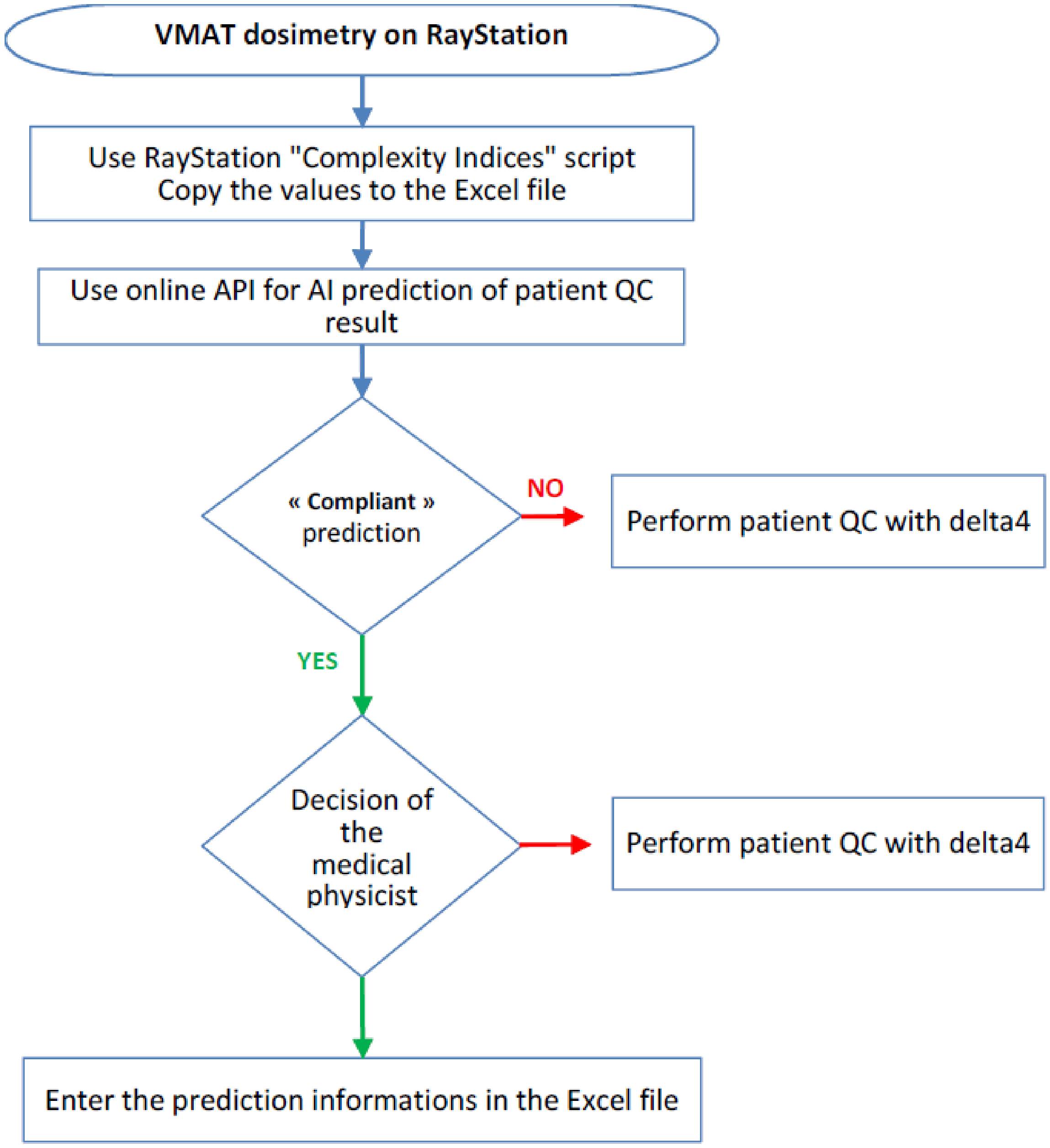

Example of workflow in clinical practice

Supp FIGURE 4:

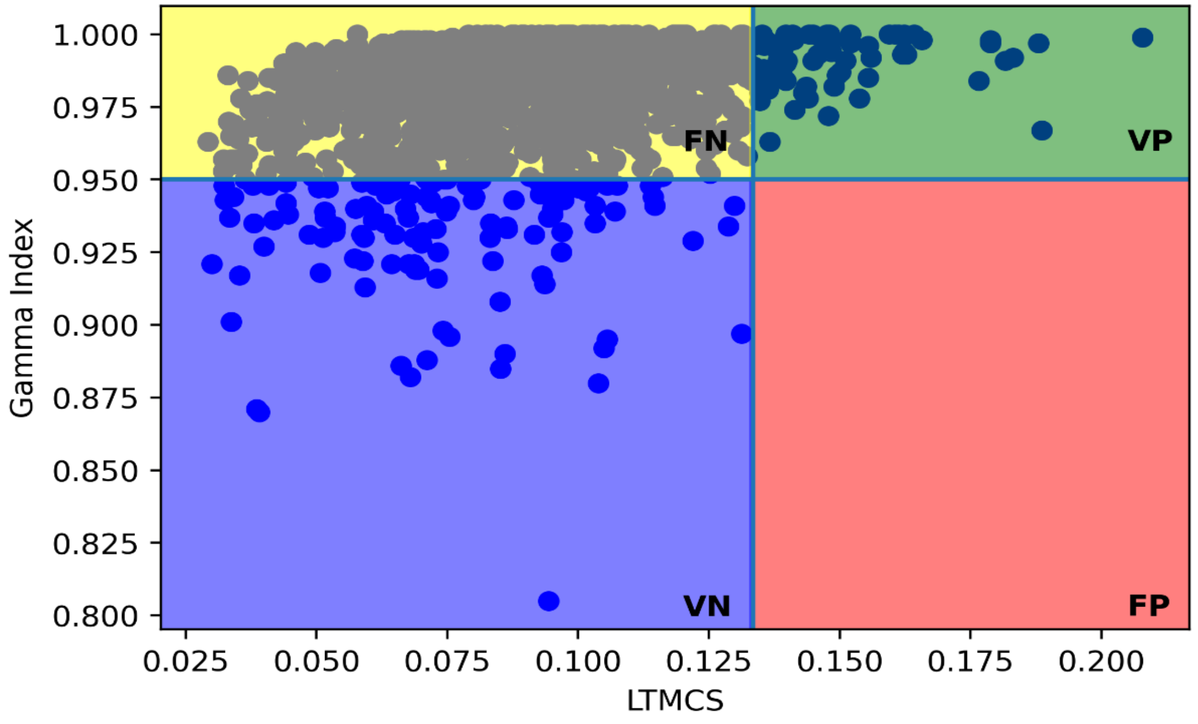

Example of using LTMCS threshold value to discriminate conformance and non-conformance QC

Supp Table 5 :

TP	TN	FP	FN	Sensitivity	Specificity
3.98 %	7.22 %	0 %	88.81 %	4.28 %	100 %

Performance of the brain specific ML model

Supp FIGURE 5:

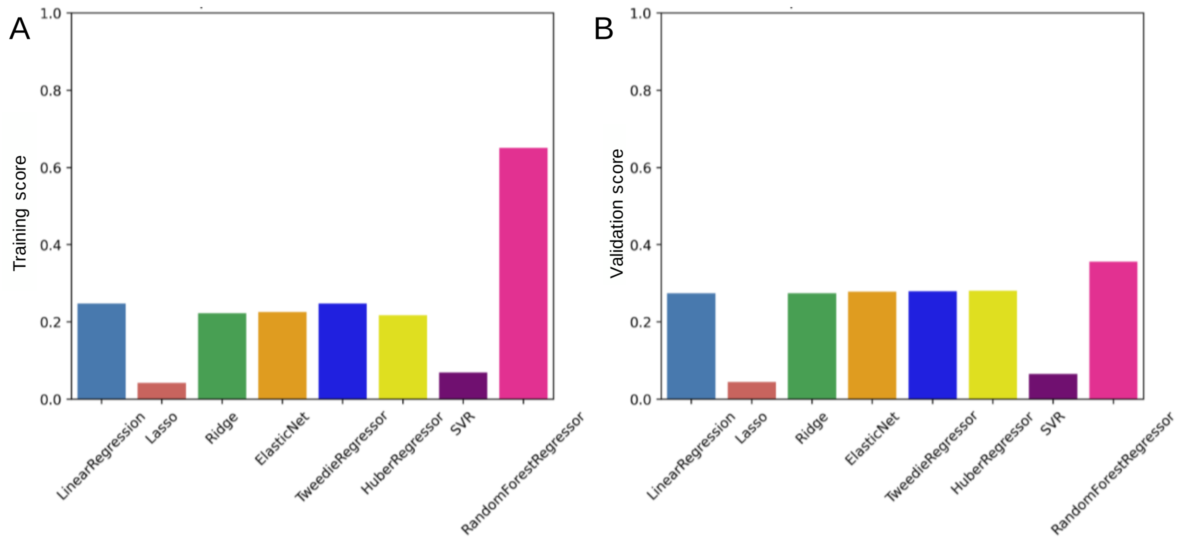

Performance of regression machine learning models, training score (A) and validation score (B)
